# Relationship between continuity of care and clinical outcomes in patients with dyslipidemia in Korea: a real world claims database study

**DOI:** 10.1038/s41598-022-06973-3

**Published:** 2022-02-23

**Authors:** Juhee Lee, Eunyoung Choi, Eunjung Choo, Siachalinga Linda, Eun Jin Jang, Iyn-Hyang Lee

**Affiliations:** 1 Department of Statistics, Kyungpook National University, Daegu, 41566 Republic of Korea; 2 College of Pharmacy, Yeungnam University, Gyeongsan, 38541 Republic of Korea; 3 Department of Pharmacy, Ulsan University Hospital, Ulsan, 44033 Republic of Korea; 4 College of Pharmacy, Ajou University, Suwon, 16499 Republic of Korea; 5 Department of Information Statistics, Andong National University, Andong, 36729 Republic of Korea; 6 Department of Health Sciences, University of York, York, YO10 5DD UK

**Keywords:** Health care, Health policy, Health services, Dyslipidaemias

## Abstract

Dyslipidemia is a risk factor for atherosclerotic cardiovascular disease and requires proactive management. This study aimed to investigate the association between care continuity and the outcomes of patients with dyslipidemia. We conducted a retrospective cohort study on patients with dyslipidemia by employing the Korea National Health Insurance claims database during the period 2007–2018. The Continuity of Care Index (COCI) was used to measure continuity of care. We considered incidence of atherosclerotic cardiovascular disease as a primary outcome. A Cox's proportional hazards regression model was used to quantify risks of primary outcome. There were 236,486 patients newly diagnosed with dyslipidemia in 2008 who were categorized into the high and low COC groups depending on their COCI. The adjusted hazard ratio for the primary outcome was 1.09 times higher (95% confidence interval: 1.06–1.12) in the low COC group than in the high COC group. The study shows that improved continuity of care for newly-diagnosed dyslipidemic patients might reduce the risk of atherosclerotic cardiovascular disease.

## Introduction

Dyslipidemia refers to imbalance in the levels of one or more kinds of lipids such as total cholesterol, low-density lipoprotein cholesterol, triglycerides and high-density lipoprotein cholesterol in the blood^1^, and is a well-known risk factor for atherosclerotic cardiovascular disease (ASCVD)^2,3^. ASCVD is increasing worldwide and is among the leading causes of mortality and morbidity. Since dyslipidemia has been reported to be a leading cause of ASCVD, early management of dyslipidemia is a prerequisite to preventing ASCVD^3–7^. Several guidelines suggest that the management of lipid modification in the presence of dyslipidemia is important^3,7,8^. However, dyslipidemia seldom has distinct subjective symptoms nor induce discomfort that patients may be aware of^9^, thus, the awareness of the disease is low^10^, resulting into rates of treatment initiation and adherence to be low. Although health behavior intervention is highly recommended^7^, self-management is likely to be ineffective due to lack of patient awareness or commitment^11^. Accordingly, to reduce obstacles to treatment for silent chronic diseases like dyslipidemia, the role of medical staff can be significant^12^. In this regard, the existing literature suggests that continuity of care (COC) between health providers and patients in chronic conditions might improve clinical outcomes^13–16^. COC is defined as ‘the degree to which a series of discrete healthcare events is experienced as coherent, and connected and consistent with the patient's medical needs and personal context’^17^. The literature indicates that improved continuity of care might lower mortality^15^, hospitalization rates^16^, emergency department costs^13^, and medical expenses^14^ in different kinds of care settings or diseases.

Some studies have shown that high COC could reduce hospitalization rates, emergency room visits, and medical costs in hypertensive^18^ or heart failure patients^19^. However, little is known about the impact of COC in patients with dyslipidemia although the burden of dyslipidemia is growing. Among South Koreans older than thirty years, 4 out of 10 were diagnosed with dyslipidemia during the 2010s^20,21^. The prevalence of hyperlipidemia, a subtype of dyslipidemia, doubled from 13.4% in 2010 to 21.4% in 2019^22^. Between 2010 and 2019, deaths from hyper-LDL-cholesterolemia increased from 12,000 to 15,000 in South Korea and from 3.75 million to 4.40 million worldwide among those older than thirty^23^. Thus in this study, we investigated the association between COC and the outcomes of patients with dyslipidemia. It was hypothesized that a high COC would be related with positive clinical outcomes in dyslipidemic patients.

## Methods

### Data sources

Claims data for analysis, which included all patients (n = 1,590,501) newly diagnosed with dyslipidemia in 2008, were provided by the Korean National Health Insurance Service (NHIS). The Korea National Health Insurance database contains all claims data for the Korean residents covered by National Health Insurance (NHI) and Medical Aid (MAid)^24^. The NHIS claims data are provided to academic researchers after de-identification of personal information. The data for this study contained de-identified patient demographic information, diagnoses, diagnostic tests, utilization of medical facilities and prescriptions of patient, death records and level of NHI contributions between 2006 and 2018^25^.

### Study design and timing

**Figure 1:**
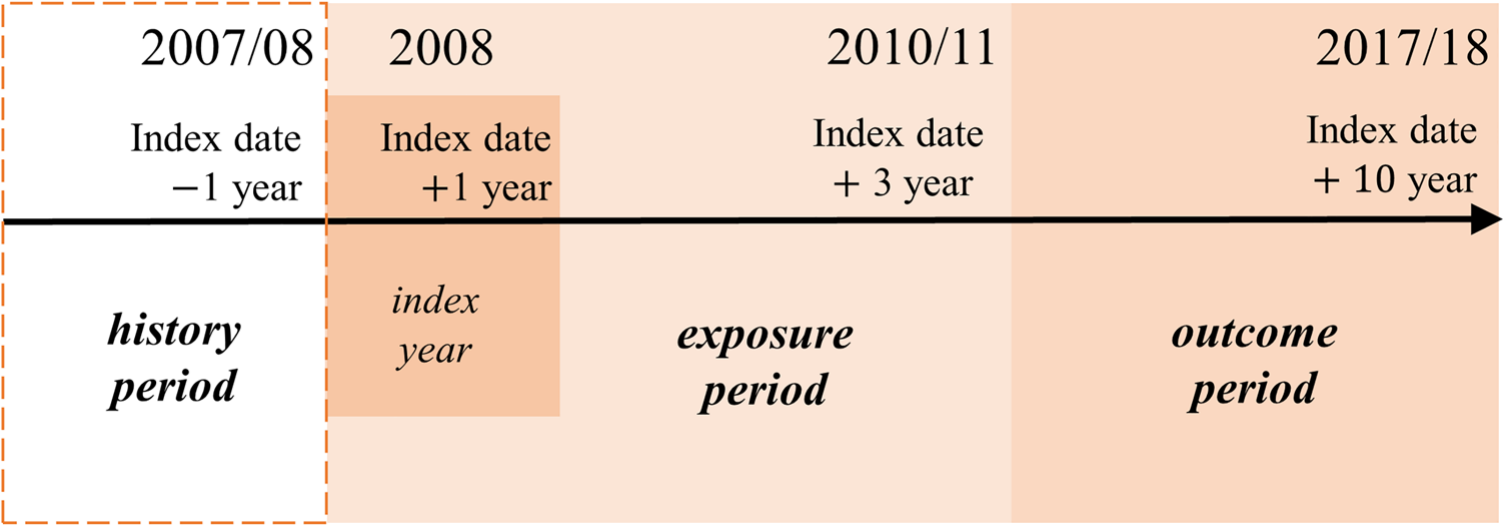
The schematic diagram of the time frame for study.

This is a retrospective cohort study. This study was conducted in accordance with the STROBE guideline^26^. Figure 1 displays the study period between 2007 and 2018 and explains each period. We set 2008 as the index year for defining the patients with dyslipidemia. The index date was set as the earliest date in 2008 of dyslipidemia diagnosis for a patient identified for this study. The history period was defined as 1 year before the index date. To avoid time-dependent bias, we independently set the exposure and outcome period in the primary analysis. The exposure period was defined as the first 3 years after the index date and the outcome period was defined as the following 7 years. We restructured available data on an annual basis from the index date. As a result, the same duration was applied to all study subjects, although the actual observation period varied. For example, in the case of a patient identified on 1 January 2008, the observation period was from 1 January 2008 to 31 December 2017, whereas for a patient identified on 1 May 2008 the observation period continued until 30 April 2018. Each patient was followed from the end of the exposure period until death, an event, or end of data availability, whichever occurred first.

### Study population

**Figure 2:**
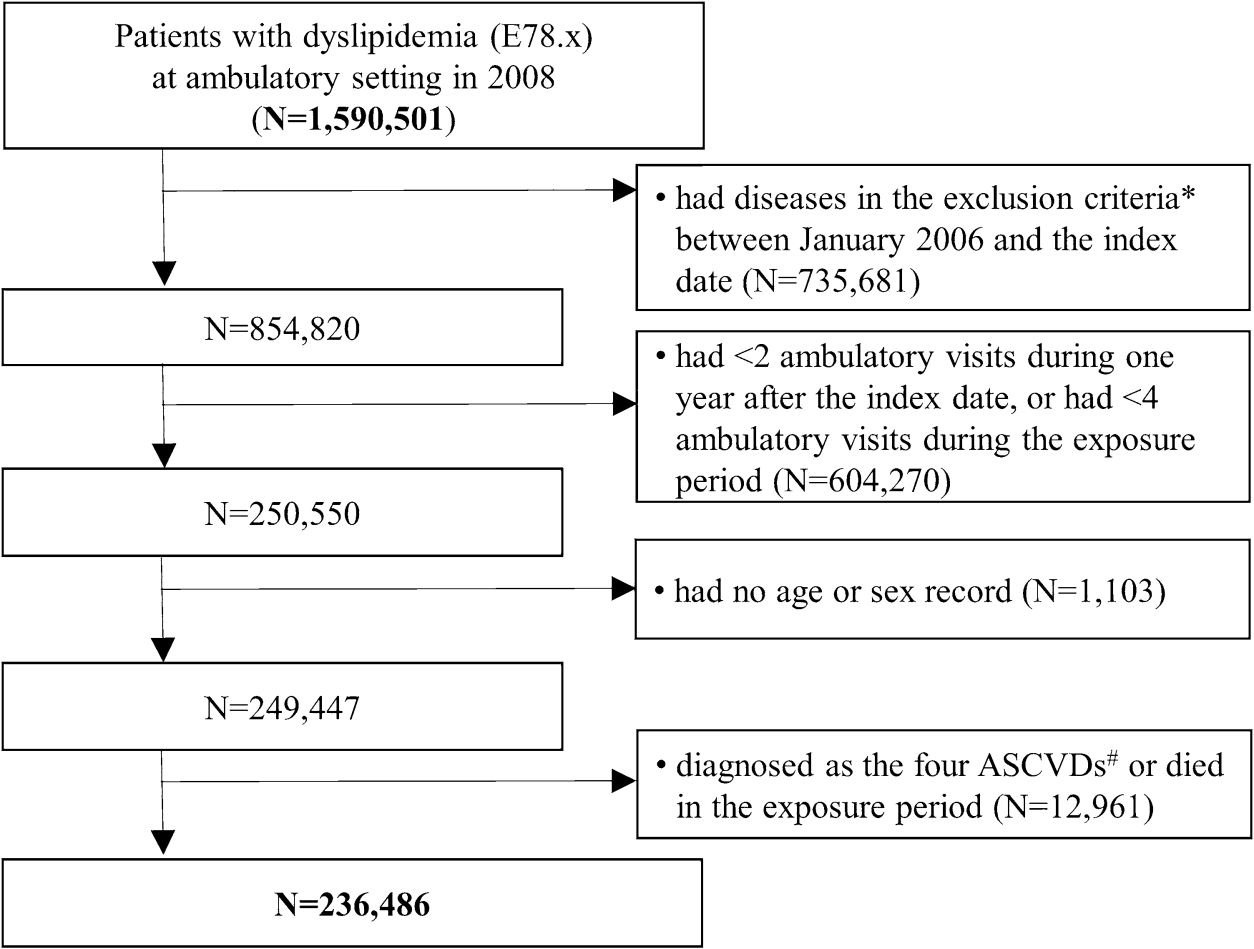
Selection of study population. ^*^Diseases in the exclusion criteria included hypertensive diseases (I10 ~ I13, I15), ischemic heart diseases (I20 ~ I25), cerebrovascular diseases and related syndromes (I60 ~ I69, G45 ~ G46), diabetes mellitus with circulatory complications (E10.5, E11.5, E12.5, E13.5, E14.5), and cancer (C00 ~ C97). ^#^The four ASCVDs included myocardial infarction (I21.0–4, I21.9, I22.0–1, I22.8–9), stable or unstable angina (I20), ischemic stroke (I63.0–6, I63.8–9), and transient ischemic attack (G45.0–3, G45.8–9).

We identified patients newly diagnosed with dyslipidemia, which was coded as E78.0 ~ E78.9 according to the International Classification of Diseases, 10th version (ICD-10)^27^, that made at least two ambulatory visits during the one year after the index date, and at least four visits during the first three years (the exposure period)^16,28,29^. Those diagnosed with hypertensive diseases (coded I10 ~ I13, I15), ischemic heart disease (I20 ~ I25), cerebrovascular diseases and related syndromes (I60 ~ I69, G45 ~ G46), diabetes mellitus with circulatory complications (E10.5, E11.5, E12.5, E13.5, E14.5) and cancer (C00 ~ C97) from January 1, 2006 to the index date were excluded because such diseases could either be related with the study outcome or affect healthcare use behavior. Those with missing personal information (e.g., age or sex) were 1,103 (0.44%) and excluded from the data. Those diagnosed with myocardial infarction (I21.0–4, I21.9, I22.0–1, I22.8–9), stable or unstable angina (I20), ischemic stroke (I63.0–6, I63.8–9), and transient ischemic attack (G45.0–3, G45.8–9), or died during the exposure period were also excluded. The selection process of study population is shown in Fig. 2.

### Measures

#### Continuity of care

To measure COC as an exposure variable, we used the Bice-Boxerman Continuity of Care Index (COCI)^28^, which is recommended for use in the context of South Korea, where patients may potentially contact many different healthcare providers. The equation used to calculate COCI is as follows:

$$\mathrm{COCI }= \frac{{\sum }_{j=1}^{M}{n}_{j}^{2}-N}{N(N-1)},$$

where $N$ is the total number of ambulatory care visits, ${n}_{j}$ is the number of visits to doctor $j$, and $M$ is the total number of doctors that a patient has met. The COC index ranges from 0 to 1. If all of the visits made by a patient are to the same doctor, the index equals 1. If each visit involves a different doctor, the index equals 0. Thus, a higher COCI indicates better continuity of care. We regarded a visit to the same doctor’s office as a visit to the same doctor in a primary care setting and a visit to the same medical department in the same hospital as a visit to the same doctor in a secondary care setting.

#### Continuity cohort

**Figure 3:**
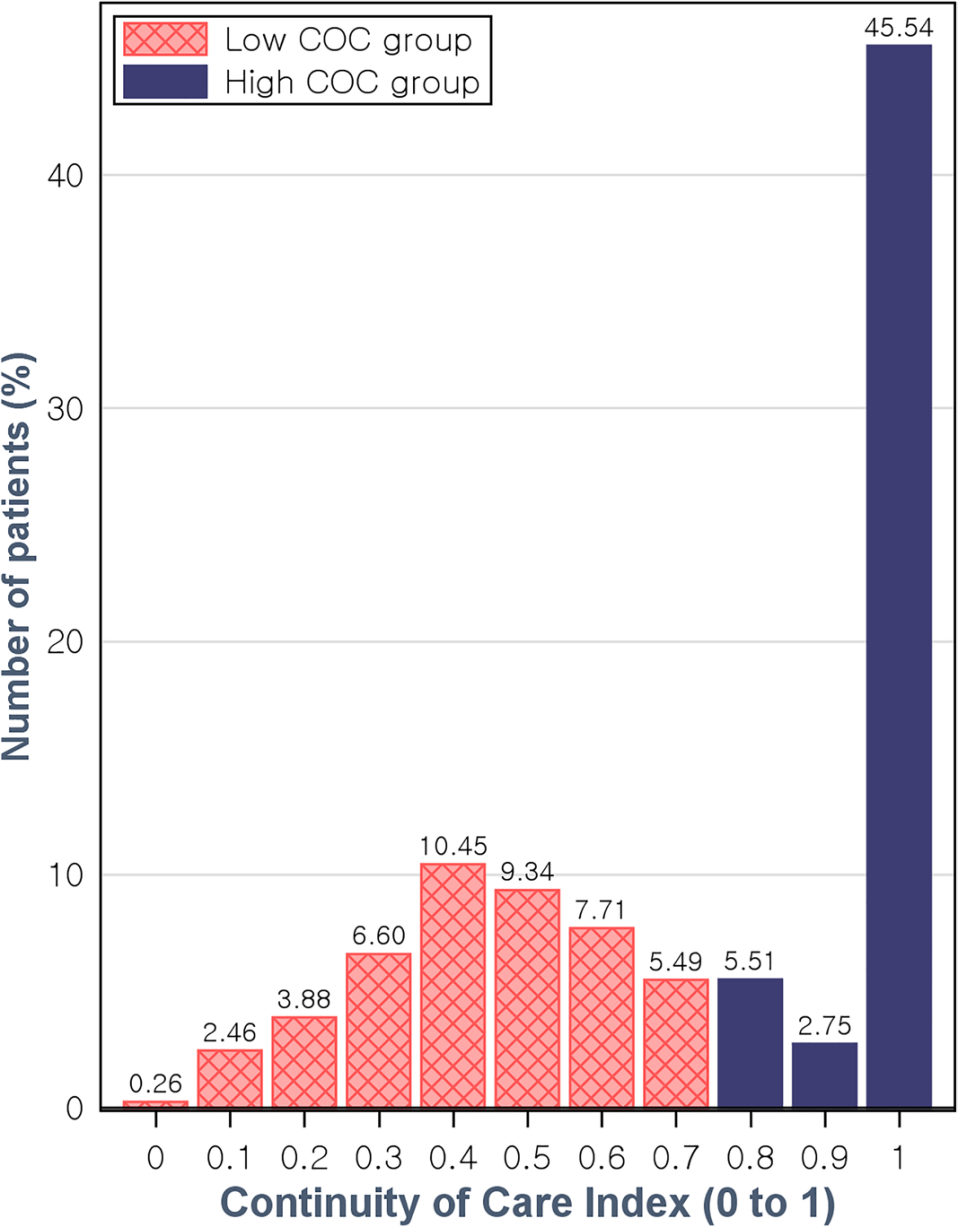
Continuity of Care Index distribution during the first 3 years. *COC* continuity of care.

As far as we are aware, there is no generally used cut-off value for high and low COCI. Thus, we set the cut-off at 0.8 and defined a COCI of ≥ 0.8 as high and a COCI of < 0.8 as low based on the first 3-year COCI distribution of our study subjects from an interim analysis. In the interim analysis, patients with a COCI of ≥ 0.8 accounted for 53.8% of all study subjects (Fig. 3). The COCI would be 0.8, if a patient made ten doctor visits that included nine visits to the same doctor.

#### Outcomes

We defined a primary outcome as the diagnosis of one of the four diseases, namely, myocardial infarction, stable or unstable angina, ischemic stroke, and transient ischemic attack during the outcome period. These ASCVDs were operationally defined as; (i) myocardial infarction, diagnosed I21.0–4, I21.9, I22.0–1, I22.8–9 and treated using Percutaneous Coronary Intervention (PCI), Coronary Artery Bypass Surgery (CABG), or fibrinolytics, (ii) stable or unstable angina, diagnosed I20 and treated using PCI, CABG, or fibrinolytics, (iii) ischemic stroke, diagnosed I63.0–6, I63.8–9 by brain imaging (CT or MRI), and (iv) transient ischemic attack, diagnosed G45.0–3, G45.8–9 by brain imaging (CT or MRI).

Secondary outcomes were defined as health services utilization and related medical costs. Health services utilization included the number of patients with one of the four ASCVD diseases, related hospitalization and their average length of hospitalization, and number of patients visiting the emergency departments (EDs) due to one of the four ASCVD diseases and their average frequency. Related medical costs included expenses incurred by dyslipidemia and the four ASCVDs and were classified as public and out-of-pocket expenditures.

#### Covariates

Covariates included individual characteristics such as sex, age, insurance contributions, payer, and urbanization level of residence at the index date. Insurance contributions were classified into three categories (high, moderate, and low). In Korea, there are two types of payers: NHI and MAid and about 97% of the population is covered by NHI and 3% by MAid^30^. The urbanization level was classified into three categories (large urban, small urban, and rural). In addition, Elixhauser comorbidity indices (ECIs) were computed as a proxy of patient health status^31^ based on diagnoses from outpatient and inpatient records during the history period. In addition, we also considered whether patients had a diagnosis of diabetes or were prescribed antihyperlipidemic agent. For reference purposes, we also collected baseline data about health services utilization and costs regardless of the disease diagnosis.

### Statistical analysis

Patient characteristics, health services utilization at baseline, COCI, and study outcomes are summarized as means and standard deviations (SDs) or medians and interquartile ranges (IQRs) for continuous variables and as frequencies and percentages for categorical variables. To compare patient characteristics by level of continuity of care, we performed the Kruskal–Wallis test for continuous variables because the assumption of normality was not satisfied. The Chi-squared test was performed to analyze categorical variables.

The association between COC and health outcome was investigated using the Cox proportional hazard regression model. The proportional assumption of the Cox proportional hazards model was checked by examining the cumulative Martingale residuals plots and the Kolmogorov-type supremum test. A Kaplan–Meier survival plot of the four ASCVDs was constructed and the p-value from the log-rank test was presented. Unadjusted hazard ratios (HRs) and adjusted HRs (adjusted for age, sex, insurance contribution group, living area, Elixhauser comorbidity index, and antihyperlipidemic agent use) were presented with 95% confidence intervals (CIs). Individuals whose information about insurance contribution and living area were missing were not excluded, and missing value was considered as an independent category in the statistical regression model. For sensitivity analysis, COCI was considered as a time-dependent exposure in the additional model, as the initial COC is likely to change over time. The subgroup analyses for the association between COC and health outcome were performed according to patient characteristics and the number of visits during the first 3 years.

The statistical analysis was performed using the SAS statistical software package (version 9.4, SAS Institute, Cary, NC, USA) and a p-value $<0.05$ was considered statistically significant.

### Ethics declarations

This study was performed in accordance with the Declaration of Helsinki and was approved by the Institutional Review Board of Yeungnam University (YU201804004002). Written informed consent was waived because this study analyzed anonymous claims data provided for research purposes by the Korean National Health Insurance Service.

## Results

### Study population

Table 1 shows the baseline characteristics of patients by continuity group. The study population included 236,486 patients, of which 53.8% were in the high COC group and 46.2% were in the low COC group. Generally, although there were more women in both groups, women were more prevalent in the low COC group (58.13%). There was relatively a small difference in the proportion of men and women in the high COC group (48.30% vs. 51.70%). Those in the low COC group were slightly older by about 1.5 years on average (p < 0.001) and more likely to reside in rural areas (9.39% vs. 8.10%). While insurance contributions and payer were significantly different (p < 0.001), the absolute differences between the groups were < 1%.

**Table 1:** Patient characteristics by level of continuity of care.

Variable		High COC group (n = 127,238)	Low COC group (n = 109,248)	*p*-value
**General characteristics**				
Sex	Women	65,778 (51.70)	63,502 (58.13)	< 0.001
	Men	61,460 (48.30)	45,746 (41.87)	
Age	Median (IQR)	50 (42–58)	51 (44–59)	< 0.001
	Mean ± SD	49.87 ± 12.41	51.27 ± 11.65	
	≥ 65 years	15,500 (12.18)	14,508 (13.28)	< 0.001
Insurance contributions^a^	High	58,746 (46.17)	49,443 (45.26)	< 0.001
	Moderate	36,809 (28.93)	30,921 (28.30)	
	Low	24,660 (19.38)	22,084 (20.21)	
Payer	NHI	121,803 (95.73)	104,059 (95.25)	< 0.001
	MAid	5,435 (4.27)	5,189 (4.75)	
Urbanization level of residence^b^	Large urban	63,655 (50.03)	54,045 (49.47)	< 0.001
	Small urban	53,151 (41.77)	44,820 (41.03)	
	Rural	10,310 (8.10)	10,262 (9.39)	
Elixhauser comorbidity index	Median (IQR)	1 (0–2)	1 (0–2)	0.110
	0	56,976 (44.78)	48,755 (44.63)	< 0.001
	1	38,029 (29.89)	32,617 (29.86)	
	2	20,122 (15.81)	16,857 (15.43)	
	3 +	12,111 (9.52)	11,019 (10.09)	
Comorbidity	Diabetes mellitus	19,350 (15.21)	12,875 (11.79)	< 0.001
	None	107,888 (84.79)	96,373 (88.21)	
Antihyperlipidemic agent use	Yes	38,484 (30.25)	34,962 (32.00)	< 0.001
	No	88,754 (69.75)	74,286 (68.00)	
**Health services utilization for all diseases (annual average during the exposure period)**				
Ambulatory visits	Median (IQR)	16.33 (10.33–25.67)	19 (11.67–30.00)	< 0.001
	Mean ± SD	20.91 ± 18.19	24.36 ± 20.93	
	4–8	51,954 (40.83)	50,175 (45.93)	< 0.001
	9–14	27,105 (21.30)	24,418 (22.35)	
	15–23	21,648 (17.01)	19,137 (17.52)	
	24 +	26,531 (20.85)	15,518 (14.20)	
Pharmacy visits	Median (IQR)	13.33 (8.33–20.33)	14.67 (9.33–22.67)	< 0.001
	Mean ± SD	15.83 ± 11.55	17.77 ± 12.95	
Annual average all medical costs (1000 KRW)^c^	Overall, median (IQR)	1119 (654–1810)	1200 (714–2002)	< 0.001
	Public expenditure	762 (440–1263)	820 (481–1411)	< 0.001
	Out-of-pocket payment	327 (181–533)	349 (200–574)	< 0.001
	Overall, mean ± SD	1566 ± 2182	1732 ± 2229	
	Public expenditure	1147 ± 1917	1275 ± 1915	
	Out-of-pocket payment	417 ± 388	454 ± 427	

*COC* continuity of care, *IQR* interquartile range, *MAid* Medical Aid, *NHI* National Health Insurance, *SD* standard deviation.

^a^Information for insurance contribution was missing for 7023 (5.5%) patients in the high COC group and 6800 (6.2%) patients in the low COC group.

^b^Information for urbanization level of residence was missing for 122 (0.1%) patients in the high COC group and 121 (0.1%) patients in the low COC group.

^c^1 US dollar = 1200 KRW in Aug 2021.

*P*-values were calculated by Kruskal–Wallis tests for continuous variables and by Chi-squared tests for categorical variables.

Average ECIs were similar in the two study groups, however, patients with 3 or more comorbidities were more likely to be in the low COC group and those with diabetes were more likely to be in the high COC group. Patients on antihyperlipidemic agents in the index year were more likely to be in the low COC group. During the exposure period, patients in the low COC group used healthcare services more frequently than those in the high COC group.

### Changes in continuity of care index

COCI decreased with time in both groups (Table 2). As the number of years increased from 3 to 10 years, the change in COC value was greater in the high COC group (1 to 0.83) compared to the low COC group (0.49 to 0.43). The numbers of visits made in 3 and 10 years were greater in the high COC group and about three quarters of these visits were to primary care.

**Table 2:** COCI and number of visits to medical facilities by period.

Variable		High COC group n = 127,238	Low COC group n = 109,248	*p*-value
**Exposure period (3 years)**				
COCI in all population	Median (IQR)	0.87 (0.50–1.00)		
	Mean ± SD	0.75 ± 0.28		
COCI by group	Median (IQR)	1.00 (1.00–1.00)	0.49 (0.36–0.60)	< 0.001
	Mean ± SD	0.98 ± 0.05	0.48 ± 0.17	
No. of visits	Median (IQR)	11 (6–21)	9 (6–17)	< 0.001
	Mean ± SD	15.40 ± 14.50	13.31 ± 12.57	
	Primary care	11.97 ± 15.65	10.14 ± 12.81	
	Secondary care	3.43 ± 6.60	3.18 ± 5.23	
**Exposure plus outcome period (10 years)**				
COCI in all population	Median (IQR)	0.58 (0.39–0.90)		
	Mean ± SD	0.62 ± 0.27		
COCI by group	Median (IQR)	0.83 (0.51–1.00)	0.43 (0.30–0.61)	< 0.001
	Mean ± SD	0.75 ± 0.25	0.47 ± 0.21	
No. of visits	Median (IQR)	33 (12–64)	34 (14–61)	0.002
	Mean ± SD	44.62 ± 44.34	43.12 ± 41.28	
	Primary care	33.94 ± 45.07	32.49 ± 40.65	
	Secondary care	10.68 ± 20.02	10.63 ± 18.08	

*COC* continuity of care, *COCI* continuity of care index, *IQR* interquartile range, *SD* standard deviation.

*P*-values were calculated by Kruskal–Wallis tests for continuous variables.

### Risk for atherosclerotic cardiovascular diseases

Table 3 shows the risks for the four ASCVDs in the two study groups. After adjusting for covariates, the adjusted hazard ratio for the risk of the four ASCVDs was 1.09 (95% CI 1.06–1.12) in the low COC group compared to the high COC group. In the sensitivity analysis using COC as a time-dependent exposure, the adjusted hazard ratio for the risk of the four ASCVDs was 1.27 (95% CI 1.23–1.31) in the low COC group compared to the high COC group.

**Table 3:** Risks for the four ASCVDs from Cox’s hazard regression models in Korean dyslipidemia patients.

Outcomes	High COC group (n = 127,238)	Low COC group (n = 109,248)	*p*-value
**Risk for the four ASCVDs**			
No. of patients having the four ASCVDs (%)	6971 (5.48)	7279 (6.66)	< 0.001
**Hazard ratio by standard Cox’s hazards regression model**			
Unadjusted HR (95% CI)	1.0 (reference)	1.23 (1.10–1.16)	< 0.001
Adjusted HR (95% CI)^a^	1.0 (reference)	1.09 (1.06–1.12)	< 0.001
**Hazard ratio by time-dependent model (sensitivity analysis)**			
Unadjusted HR (95% CI)	1.0 (reference)	1.30 (1.26–1.35)	< 0.001
Adjusted HR (95% CI)^a^	1.0 (reference)	1.27 (1.23–1.31)	< 0.001

*ASCVD* atherosclerotic cardiovascular disease, *CI* confidential interval, *COC* continuity of care, *HR* hazard ratio, *IQR* interquartile range, *SD* standard deviation.

^a^The adjusted HR was analyzed after adjusting for covariates including sex, age, insurance contribution, payer, urbanization level of residence, comorbidity as Elixhauser score and antihyperlipidemic agent use.

**Figure 4:**
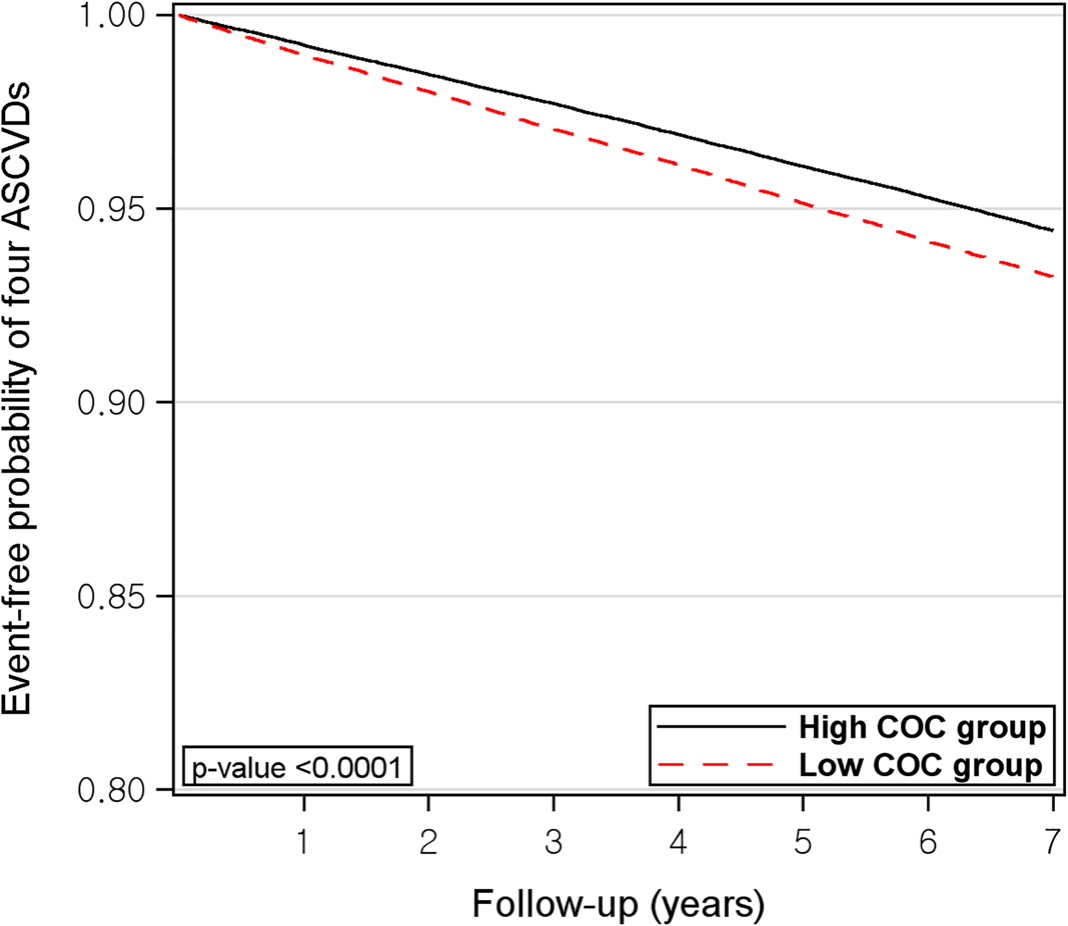
Event-free probability of four ASCVDs. *ASCVD* atherosclerotic cardiovascular disease, *COC* continuity of care.

In Fig. 4, during the 7-year follow-up period, the probability of being event-free of the four ASCVDs were significantly lower in the low COC group (p < 0.001).

### Related health services utilization and costs

Patients in the low COC group were more likely to be hospitalized or visit an ED due to one of the four ASCVDs and to have greater medical costs than those in the high COC group (Table 4). The frequency of ED visits per patient among those who had events was not significantly different between groups.

**Table 4:** Health services utilization and related medical costs by level of continuity of care.

Outcomes	High COC group (n = 127,238)	Low COC group (n = 109,248)	*p*-value
**Health services utilization due to the four ASCVDs**			
No. of patients hospitalized (%)	5237 (4.12)	5375 (4.92)	< 0.001
Average length of hospitalization days per patient per year, median (IQR)	10 (4–24)	9 (3–23)	0.001
No. of patients visiting ED (%)	2893 (2.27)	2853 (2.61)	< 0.001
Frequency of visiting ED per patient per year, median (IQR)	1 (1–1)	1 (1–1)	0.558
**Related medical costs**			
Disease related medical costs^#^ 1000 KRW per patient per year, median (IQR)	89 (20–208)	100 (31–229)	< 0.001
Public expenditure	58 (13–141)	66 (20–160)	< 0.001
Out-of-pocket payment	24 (5–62)	27 (7–66)	< 0.001
Disease related medical costs^#^ 1000 KRW per patient per year, mean ± SD	315 ± 1460	347 ± 1439	
Public expenditure	249 ± 1250	275 ± 1236	
Out-of-pocket payment	65 ± 249	71 ± 247	

*ASCVD* atherosclerotic cardiovascular disease, *CI* confidential interval, *COC* continuity of care, *ED* Emergency department, *HR* hazard ratio, *IQR* interquartile range, *SD* standard deviation.

^a^Diseases related medical costs included costs for treating dyslipidemia and dyslipidemia related four ASCVDs; 1 US dollar = 1200 KRW in Aug 2021.

*P*-values were calculated by Kruskal–Wallis tests for continuous variables, and Chi-squared tests for categorical variables.

### Subgroup analysis

**Figure 5:**
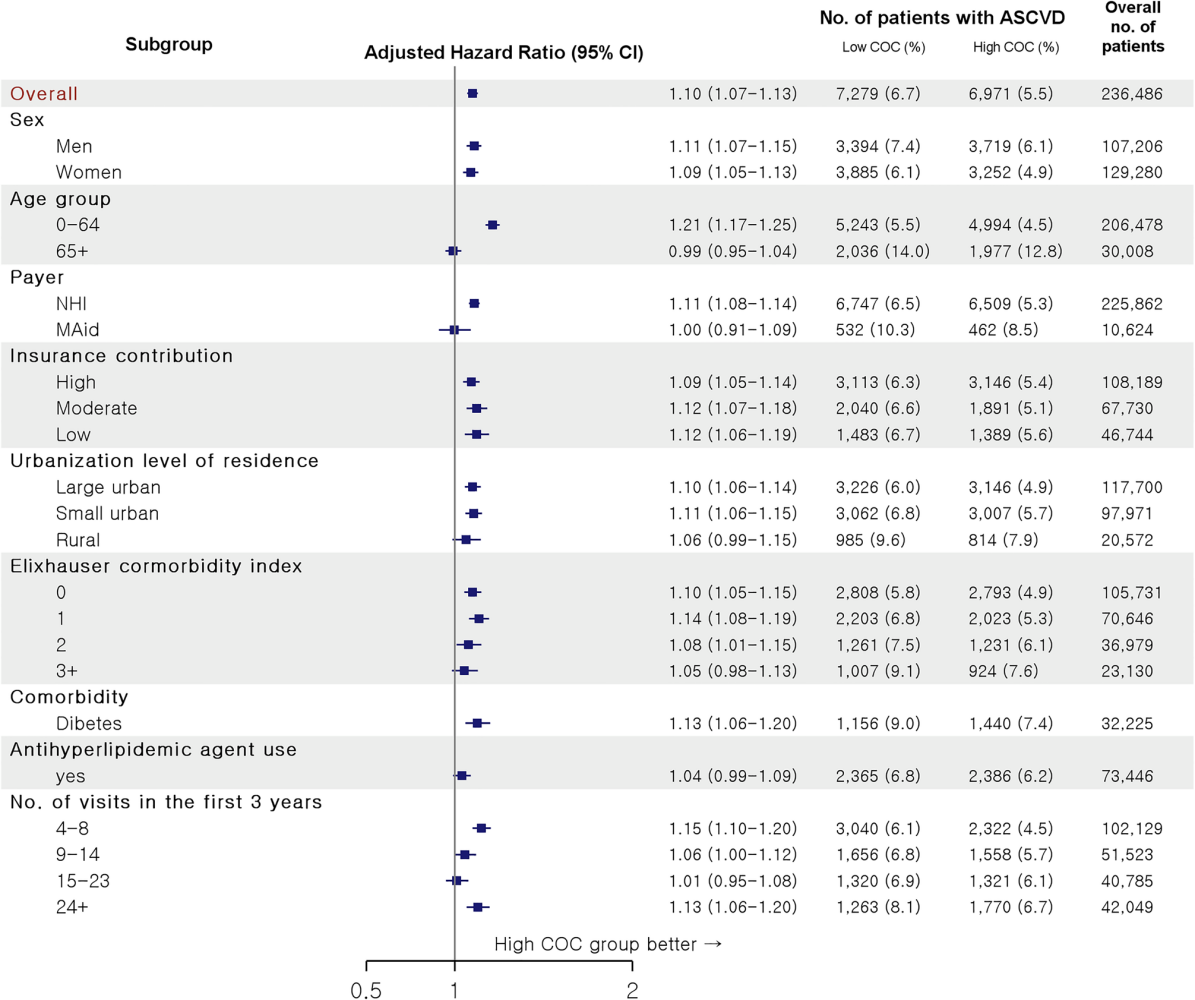
Subgroup analysis for the association between continuity of care and the risk for the four ASCVDs. *ASCVD* atherosclerotic cardiovascular disease, *CI* confidence interval, *COC* continuity of care, *MAid* Medical Aid, *NHI* National Health Insurance.

The subgroup analysis demonstrated the same trend as the primary findings in regard to the risk for the four ASCVDs in most attributes (Fig. 5). However, the risk differences between the two groups were not statistically significant seemingly due to the small number of samples in the following groups; elderly, patients in MAid, those in rural area, those with ECI greater than 3, those who took antilipidemic agents, and those who made 15–23 doctor visits during the first 3 years.

## Discussion

The results of this study show that a low COC level in newly diagnosed dyslipidemic patients is associated with increased risk of myocardial infarction, stable or unstable angina, ischemic stroke, or transient ischemic attack. From the perspective of a medical staff, patients in the high COC group may be interacted with continuously, and thus, staff are more likely to provide good quality of care (e.g., patient-tailored care)^32^. From the patient's point of view, they are more likely to be satisfied with medical staff, which might improve management and inter-personal trust^33^, and probably improve clinical results.

The study also demonstrates that a high COC level might have positive impact on health resources utilization and medical costs. However, it may be difficult to merely ascribe medical costs savings to the impact of high COC since patients with low COC already had higher medical costs in the first three years. Future studies are needed to determine the actual magnitude of cost savings. In previous studies, women, the elderly^34–36^ and patients further away from medical institutions^35^ tended to change doctors more frequently, resulting in low COC levels. The same phenomena were observed in the present study, that is, the low COC group contained higher proportions of women, elderly, and patients living in rural areas. A low socioeconomic status may increase the possibility of negative outcomes by lowering the chances of receiving healthcare services^37^, though this was not obvious with patients in the low COC group of the present analysis.

In the present study, the mean COCI among study subjects during the first 3 year period was 0.75 and decreased to 0.62 in the 10 year period. While no previous study has explored COC in patients with dyslipidemia, the distributions of COCI observed in two studies on patients with cardiovascular diseases compare well with our findings. Hong et al. found the mean COCI of 0.74 in Korean patients with hypertension in a 3-year period^18^. Another study by Vogt and colleagues, investigated COC in German patients with heart failure, reported that the mean COCI was 0.77 in a 2 year period^19^. There were some differences in studies for other diseases. The mean COCI in a 2 year span was reported to be 0.83 in Korean type 2 diabetes mellitus patients^36^, and 0.65 in Taiwanese COPD patients^38^. These demonstrated that the distribution of COCI is dependent on disease, measurement period, and country even for chronic diseases.

To the best of our knowledge, this study is the first to evaluate the effects of COC on patients with dyslipidemia using long-term real world data on the Korean population. In this study, exposure and outcome period were independently set in the primary analysis to avoid time-dependent bias^39^, and the sensitivity of the hazard ratios were further tested by using COCI as a time-dependent covariate. ASCVD is a chronic disease and takes a relatively long time to progress from dyslipidemia. In this regard, the present study was sufficiently long to explore the relationship between COC and clinical outcomes as it utilized 10 years of claims data.

However, the use of secondary data for analysis inherently introduces limitations. First, though sophisticated statistical methods were used to control for potential confounders, they may have failed to exclude the effects of some meaningful factors affecting patients’ relationships with professionals such as occupation, education, or health behaviors, which are seldom included in claims data. Second, the claims data did not contain any information about individual doctors. To compensate for this, we considered visits to the same medical department in the same hospital in a secondary care setting as visits to the same provider, because most patients see the same doctor when they visit the same medical department in the same institution. Third, this study employed an empirical method to classify patients into the two continuity groups. There are no previous study suggesting a standard or optimal procedure for grouping patients based on COCI, rather median values^36,40^, tertiles^41,42^, and other metrics^43,44^ have been used. This may mean that the distributions and cut-offs of COC are research population dependent. Furthermore, the Korean healthcare system provides universal coverage, and patients have the right to choose a healthcare provider with few legal restrictions, which limits the external validity of this study.

The study findings suggest that policies improving care continuity between doctors and patients with dyslipidemia results in positive clinical outcomes. Future policies need to identify factors that hinder lasting quality relationships and make efforts to address them. Also, efforts should be made to ensure that doctors and patients are aware of the good impact of continuity of care. In this regard, qualitative study investigating the perceptions of professionals or patients on continuity of care will be helpful to disclose their awareness and views of it.

## Conclusion

Improved continuity of care might decrease the risks of myocardial infarction, stable or unstable angina, ischemic stroke and transient ischemic attack in patients with dyslipidemia, and thus, this would reduce utilization of costly healthcare services and relevant costs.

## Data Availability

The raw data that support the findings of this study are available only for authorized researchers in South Korea and for a limited period due to the information protection law for patient privacy.
